# Comparison of patient-reported outcomes and healthcare utilization between metastatic colorectal cancer patients receiving systemic therapy with or without concurrent palliative care support: a retrospective cohort study

**DOI:** 10.3389/fonc.2026.1882581

**Published:** 2026-09-03

**Authors:** Xiaoli Liu, Fangtuan Wu, Lunlan Li

**Affiliations:** 1 School of Nursing, Anhui Medical University, Hefei, China; 2 Department of Oncology, Hefei Third People’s Hospital, Hefei, China; 3 Endoscopy Center, First Affiliated Hospital of Anhui Medical University, Hefei, China

**Keywords:** metastatic colorectal cancer, palliative care, patient reported outcome, retrospective cohort study, utilization of medical resources

## Abstract

**Background:**

Patients with metastatic colorectal cancer (mCRC) experience substantial symptom burden, yet evidence on the integration of specialist palliative care (PC) in real-world settings remains limited. We assessed the impact of concurrent PC on patient-reported outcomes and healthcare utilization.

**Objective:**

To evaluate the impact of integrating professional palliative care (PC) with standard systemic therapy on patient-reported outcomes, medical resource utilization, and overall survival (OS) in metastatic colorectal cancer (mCRC).

**Methods:**

This retrospective cohort study included 201 mCRC patients receiving systemic therapy (Jan 2023–Jan 2025), of whom 69 received concurrent multidisciplinary PC (combined group) and 132 received standard care alone (non-combined group). We evaluated pain (NRS), clinically documented clinically documented emotional distress (diagnosis or medication use), treatment goal consistency, unplanned hospitalizations, emergency visits, end-of-life chemotherapy, and OS. The study was approved by the institutional ethics committee, which waived the requirement for informed consent due to the retrospective design. Statistical analyses used chi-square, Mann-Whitney U, and Cox regression. Two-sided P<0.05 was significant.

**Results:**

At baseline, the combined group was older (median age higher, P = 0.002), had a greater proportion with ECOG PS≥2 (P = 0.008), and had a higher Charlson comorbidity index (P<0.001). Despite these disadvantages, they reported significantly greater pain reduction (P<0.001), lower clinically documented emotional distress rates (P = 0.007), and higher treatment goal consistency (P<0.001). Unplanned readmissions, emergency department visits, and chemotherapy within 30 days of death were all significantly lower (each P ≤ 0.001). Multivariate logistic regression confirmed that PC independently reduced the risk of clinically documented emotional distress (adjusted OR 0.42, P = 0.008) and increased goal consistency (OR 7.85, P<0.001). Overall survival did not differ between groups (median 19.1 vs 18.3 months; HR 0.92, P = 0.632).

**Conclusion:**

Integrating professional PC into standard mCRC management significantly enhances symptom relief, treatment goal alignment, and reduces aggressive end-of-life interventions without affecting survival. These findings strongly support the routine incorporation of multidisciplinary PC as a core component of high-quality cancer care, improving patient-centered outcomes and promoting efficient resource utilization. Early PC integration aligns with guideline recommendations and holds promise for cost-effective cancer care delivery.

## 1 Introduction

The treatment pattern of metastatic colorectal cancer (mCRC) has undergone profound changes in the past two decades, evolving from palliative treatment mainly based on chemotherapy to a comprehensive management strategy that includes targeted therapy, immunotherapy, and local intervention (1). Although these advances have significantly prolonged the survival of patients, the disease itself and the symptom burden brought by its treatment, such as pain, fatigue, clinically documented emotional distress, still seriously affect the quality of life of patients (2). In this context, the goal of cancer treatment has evolved from simply pursuing the extension of survival time to maximizing the maintenance or improvement of patients’ quality of life while prolonging survival (3). This concept has become a core consensus in clinical practice of advanced cancer (4). The effective management of symptoms is not only related to the subjective feelings of patients (5), but also to the complex pathophysiological mechanisms behind it, such as the interaction between chronic inflammation and the neuroendocrine system, which may indirectly affect the tumor microenvironment and treatment tolerance (6). Therefore, how to systematically evaluate and intervene in these symptoms constitutes a key link in optimizing the overall management of mCRC (7).

However, the traditional medical model centered on anti-tumor therapy has significant limitations in addressing the multidimensional needs mentioned above (8). Oncologists often face the pressure of tight diagnosis and treatment time, focusing on disease control (9), and may not have the time to deeply evaluate and deal with complex symptom clusters and psychosocial issues (10). In addition, communication regarding end-of-life treatment decisions, such as whether to continue high-intensity chemotherapy or transfer to the intensive care unit, is often rushed during times of crisis which may result in treatment choices that are inconsistent with the patient’s personal values and preferences (11). This inconsistency has been proven to be associated with lower quality of life for patients, higher consumption of medical resources, and heavier grief burden on family members (12). Although there are guidelines recommending early integrated palliative care for advanced cancer patients its application in real-world practice is still insufficient and mostly concentrated in the last few weeks of life, failing to fully realize its potential value in improving symptoms and assisting decision-making throughout the disease (13).

Although early palliative care has demonstrated survival and quality-of-life benefits in advanced non-small cell lung cancer, evidence specific to mCRC is sparse. Most studies in colorectal cancer are limited to mixed advanced-cancer cohorts or focus on single outcomes (14). A studied suggest that PC integration is associated with reduced symptom burden and less aggressive end-of-life care in gastrointestinal cancers, but robust, real-world data evaluating patient-reported outcomes and treatment-goal concordance concurrently are lacking (15). Our study addresses this gap.

This study aims to fill the knowledge gap mentioned above through a retrospective cohort analysis. We assume that, based on standard systemic therapy, early integration of specialized palliative care support provided by multidisciplinary teams can more effectively improve pain and emotional symptoms in mCRC patients, enhance consistency between treatment decisions and patient goals, and guide the formation of a more rational and patient friendly medical resource utilization model, without sacrificing overall survival. Through in-depth analysis of real-world data, this study aims to provide more convincing empirical evidence for promoting a patient-centered and integrated palliative care comprehensive treatment model in the mCRC and wider population of advanced solid tumors.

## 2 Data and methods

### 2.1 General information

**Figure 1:**
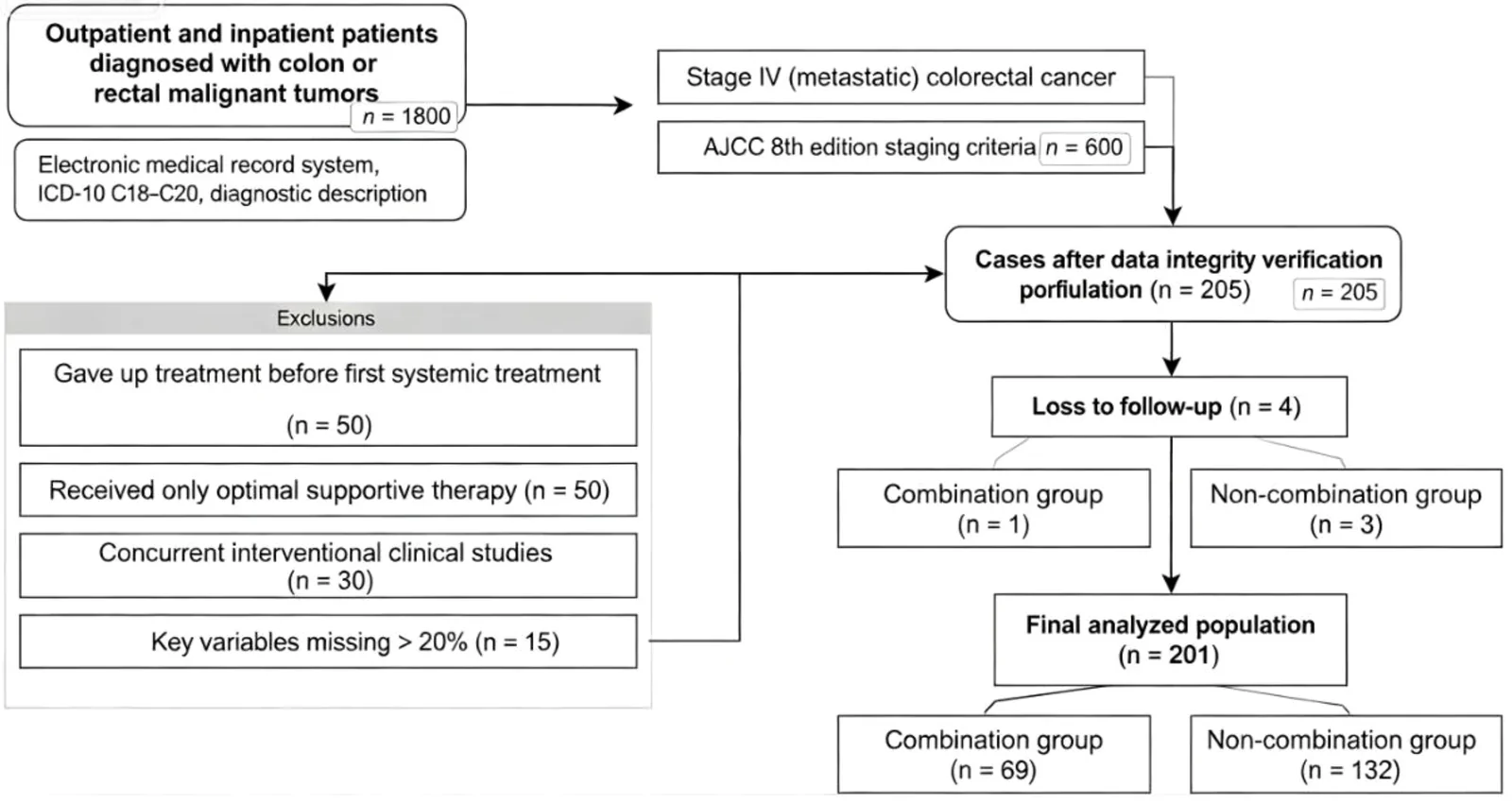
Patient study flow chart.

This study adopted a retrospective design and continuously screened all patients with metastatic colorectal cancer (mCRC) diagnosed and receiving systemic anti-tumor therapy in our oncology department from January 1, 2023 to January 31, 2025, using our hospital’s electronic medical record system and hospice specialty electronic record module. Based on qualified cases with complete data available during the research period, we adopted a full sample inclusion strategy. Based on the preliminary estimation of the hospice consultation rate in our hospital, we expect to include approximately 205 eligible patients within a 24 month admission and discharge cycle. According to our hospital’s previous clinical practice model, it is expected that the proportion of patients who receive professional palliative care team intervention during systematic treatment is about 35% (n = 70), while the proportion of patients who do not receive this service as a control is about 65% (n = 135). This grouping ratio is consistent with the reality in the real world that the penetration rate of palliative care in advanced cancer patients is gradually increasing but has not yet become routine. The screening and inclusion process of patients is detailed in **Figure 1**.

### 2.2 Inclusion and exclusion criteria

#### 2.2.1 Inclusion criteria

1. Age is 18 years old and above, with no gender restrictions.
2. The diagnosis of colon or rectal adenocarcinoma was confirmed through histopathological examination, based on the pathological report of surgical specimens or endoscopic biopsy tissue.
3. According to the 8th edition of the American Joint Committee on Cancer (AJCC) Tumor Staging Manual, distant metastasis is confirmed by imaging examinations (such as enhanced CT, MRI, or PET-CT) during initial diagnosis or subsequent follow-up, and the disease is classified as stage IV.
4. During the research window period from January 1, 2023 to January 31, 2025, at least one complete cycle of systemic anti-tumor therapy shall be initiated and completed in the Oncology Department of our hospital. The definition of systemic therapy includes but is not limited to: combination chemotherapy with fluorouracil drugs, targeted therapy based on bevacizumab or cetuximab, immune checkpoint inhibitor therapy targeting highly unstable microsatellites, and any combination of the above regimens.
5. The patient’s outpatient medical records, inpatient medical records, nursing records, laboratory and imaging reports and other key medical documents are complete and available for researchers to access and extract.

#### 2.2.2 Exclusion criteria

1. After being diagnosed with stage IV disease for the first time, patients are evaluated by the attending physician as only suitable for Best Supportive Care (BSC) before starting any systemic anti-tumor treatment. These patients usually cannot tolerate anti-tumor therapy due to extremely poor physical condition (such as ECOG PS score ≥ 3) or severe organ dysfunction.
2. During the observation period of this study, participate in any other interventional clinical research that may intervene or affect patient symptom management, quality of life assessment, treatment decision-making, or end-of-life care models. This includes but is not limited to randomized controlled trials for managing symptoms such as cancer pain, nausea and vomiting, fatigue, as well as clinical studies evaluating novel palliative interventions.
3. The key baseline variables recorded in the medical records are missing by more than 20%. The key variables specifically refer to: age, gender, primary tumor location, number and distribution of metastatic sites, baseline Eastern Cooperative Oncology Group Performance Status (ECOG PS), Charlson Comorbidity Index (CCI), and type of first-line systemic treatment plan.

Each PC consultation consisted of a structured, in-person assessment conducted by at least one palliative care specialist and one specialist nurse, following institutional guidelines adapted from the ASCO/NCCN palliative care standards. Core components included comprehensive symptom assessment and titration, psychosocial evaluation, and discussion of goals of care and advance care planning. Informal telephone contacts or brief check-ins were not classified as formal PC consultations. The exact date of first PC consultation was recorded for a subset of patients, but standardized timing data were missing for others, which prevented a formal analysis of early versus late PC integration.

### 2.3 Equipment and data sources

The data for this study was sourced from three independent in-hospital information systems:

#### 2.3.1 Medical data master platform

The hospital’s unified electronic medical record system (winex), version 6.0. This platform integrates all core medical data including outpatient, inpatient, emergency, surgical, laboratory (LIS), imaging (PACS), and pathology reports. All clinical event timestamps, diagnoses, medical orders, test results, nursing records, and course records are provided by this system.

### 2.4 Research methods

#### 2.4.1 Research design and exposure definition

This study adopts a retrospective cohort study design. The exposure was defined as receiving at least one formal, in-person multidisciplinary PC consultation during the period from initiation of first-line systemic therapy until death or the study cutoff date (April 30, 2025). Because the timing of the first PC consultation varied and was not systematically recorded in a standardized manner for all patients, a binary (ever/never) classification was used. We recognize that patients who died early had less opportunity to receive PC, which may introduce immortal time bias. To evaluate the robustness of our findings, we performed a sensitivity analysis excluding patients who died within 90 days of starting systemic therapy; the results for key outcomes remained consistent (**Supplementary Table 1**). The team’s services include but are not limited to: complex pain and symptom management, assessment and intervention of psychosocial distress, clarification and communication of treatment goals and advance care plans. The control group consisted of patients who had never received formal consultation or outpatient follow-up from the aforementioned palliative care team during the study observation period.

#### 2.4.2 Data extraction and quality control

To ensure the objectivity and accuracy of data extraction, we have designed a standardized electronic data extraction table. Two research assistants, who were blinded to the study hypothesis and received standardized training, independently extracted all data from the three aforementioned sources in a back-to-back manner. Extract content strictly following the observation indicators defined in Section 2.5. During the extraction process, any disagreement regarding the understanding of medical records shall first be resolved through consultation between two researchers; If consensus cannot be reached, it shall be submitted to a senior oncologist or hospice specialist who is not directly involved in data extraction for arbitration. After the data extraction was completed, we randomly selected 10% of the cases for secondary verification to evaluate the consistency of the data extraction.

#### 2.4.3 Bias control and statistical analysis strategies

All primary and secondary outcome analyses, the Fine-Gray competing risk model, the sensitivity analysis excluding early deaths, and the subgroup analyses were prespecified in the statistical analysis plan. Propensity score matching was added as a *post hoc* sensitivity analysis to further address baseline imbalances. Baseline balance analysis: Firstly, we will compare the differences in baseline characteristics between the combined palliative care group (exposed group) and the non-combined group (non exposed group). For continuous variables, if they follow a normal distribution, independent sample t-test is used; otherwise, Mann Whitney U test is used; For categorical variables, chi square test or Fisher’s exact test are used. All multivariable models included a pre-specified set of clinically relevant covariates: age, ECOG PS (≥2 vs. 0–1), CCI, presence of peritoneal metastasis, and first-line treatment regimen. For other baseline variables with between-group P<0.10, we tested their impact by adding them individually; they were retained in the final model only if they altered the effect estimate of PC support by more than 10%. This approach balances clinical plausibility with empirical adjustment for observed imbalance.

##### 2.4.3.1 Main analysis

A multivariate logistic regression model was used to calculate the adjusted odds ratio (aOR) and its 95% confidence interval (CI) for binary outcome measures, such as whether chemotherapy was received within 30 days before death. For continuous outcome measures such as total length of hospital stay, number of emergency visits, etc., as these data usually exhibit a right skewed distribution, we plan to use a Generalized Linear Model (GLM), select Gamma distribution as the error distribution, and use a Log link function for modeling to estimate the ratio of geometric means between the two groups and their 95% CI. All models will include baseline imbalance variables and potential confounding factors determined based on clinical experience (such as age, ECOG PS score, number of transfer sites) as covariates.

##### 2.4.3.2 Sensitivity analysis one: propensity score matching

To reduce confounding effects caused by patient selection bias, we will conduct propensity score matching analysis. A non-parsimonious logistic regression model was constructed with the dependent variable of whether or not receiving palliative care support, and all baseline covariates (including age, gender, ECOG PS, CCI, primary lesion location, number of metastatic sites, and type of first-line treatment plan) as independent variables, to calculate the propensity score for each patient. Subsequently, using the nearest neighbor matching method, the clamp value was set to a Logit value of 0.2 times the standard deviation of the propensity score, and patients in the combined palliative care group were matched with control group patients in a ratio of 1:2. After matching, the balance of baseline features between the two groups will be evaluated again, and the primary and secondary outcome measures of the matched queue will be compared.

##### 2.4.3.3 Sensitivity analysis 2

###### 2.4.3.3.1 Competitive risk analysis

For the endpoint of “receiving chemotherapy 30 days before death”, its occurrence is significantly affected by the competitive event of death. If the traditional Kaplan Meier method is used, death will be considered as a deletion, thereby overestimating the use of chemotherapy. Therefore, we will use the Fine Gray competitive risk model for analysis, treating death as a competitive event and calculating the Subdistribution Hazard Ratio (SHR) and its 95% CI to more accurately assess the impact of palliative care on end-of-life chemotherapy use.

###### 2.4.3.3.2 Subgroup analysis

To explore the heterogeneity of palliative care support effects, we will conduct subgroup analysis in the general population according to predetermined clinically relevant subgroups. Subgroups include: primary tumor site (colon vs rectum), age (<70 years vs ≥ 70 years), and baseline ECOG PS score (0–1 points vs ≥ 2 points). By introducing interaction terms into the multivariate regression model, we aim to examine whether there is a significant interaction between palliative care support and various subgroup variables.

### 2.5 Observation indicators

The primary outcomes were change in pain intensity (NRS) and treatment goal concordance. Secondary outcomes included clinically documented emotional distress, global health status/QoL, unplanned hospitalizations, emergency department visits, chemotherapy within 30 days of death, ICU admission within 14 days of death, grade ≥3 adverse events, and overall survival. The specific definitions are as follows:

#### 2.5.1 Pain control status

The Numerical Rating Scale (NRS) was used to evaluate the intensity of pain in patients. NRS is an 11 point numerical scale ranging from 0 to 10, where 0 represents’ painless’ and 10 represents’ the most severe pain imaginable ‘. We extracted the worst pain NRS scores recorded by patients at baseline (i.e. within 3 days before the start of the first systemic treatment) and during the treatment process. If the NRS score is not directly recorded in the medical record, the researcher will perform standardized conversion based on the textual description of the pain level in the nursing record (such as “mild pain”, “unbearable severe pain”), and the conversion rules have been trained for consistency before data extraction. If a numerical NRS was not directly documented, textual descriptors were converted as follows: ‘mild pain’ was coded as NRS 2, ‘moderate pain’ as NRS 5, ‘severe pain’ as NRS 8, and ‘unbearable/excruciating pain’ as NRS 10. These conversions were validated by independent review of 30 random records, with an agreement rate of 92% (κ = 0.87).

#### 2.5.2 Clinically documented emotional distress

Indirectly assess a patient’s clinically documented emotional distress through diagnostic terminology or medication use recorded in medical records. Specific definition: During the study observation period, the medical records (including psychiatric consultation records, course records, discharge summaries) clearly record diagnoses such as “anxiety state”, “depression state”, “adaptation disorder”, or at least one prescription drug for anxiety or depression (such as selective serotonin reuptake inhibitors (SSRIs), serotonin and norepinephrine reuptake inhibitors (SNRIs), benzodiazepines, etc.). This is a binary indicator based on medical records (yes/no).

#### 2.5.3 Overall quality of life assessment

For patients who have completed the European Organization for Research and Treatment of Cancer Quality of Life Core Scale 30 (EORTC QLQ-C30, version 3.0) at our hospital, we extracted their scores from the “Global Health Status/Quality of Life” subscale. This subscale includes question 29 (“How do you evaluate your overall health status over the past week?”) and question 30 (“How do you evaluate your overall quality of life over the past week?”), with each question rated on a scale of 1–7 points. According to the EORTC scoring manual, the scores of this subscale are linearly converted to a range of 0-100, with higher scores indicating better overall quality of life. Due to not all patients completing the scale, this indicator is only analyzed in the subset of patients for which data is available.

#### 2.5.4 Consistency of treatment objectives

This is a key process indicator in this study. Two researchers, who were not aware of the grouping situation, independently reviewed the conversation records of each patient’s course records regarding major treatment decisions (such as initiating second-line or third line chemotherapy, deciding to transfer to the intensive care unit ICU, choosing cardiopulmonary resuscitation), especially the meeting records of the palliative care team. Evaluate whether these key decisions are consistent with the pre care plan recorded in the medical records, known to the patient, or the overall treatment goals defined by the palliative care team (such as “prioritizing symptom control and improving quality of life” or “prioritizing life extension”). The result is judged as “consistent” or “inconsistent”. Calculate the consistency Kappa value between the evaluation results of two researchers. If the Kappa value is less than 0.6, the third senior researcher will make the final decision.

#### 2.5.5 Unplanned medical exposure

Count the unplanned outpatient or inpatient events that occurred for any reason in each patient from the beginning of the first systematic treatment until death or the study cutoff date (April 30, 2025). Specifically, it includes: the number of emergency department visits (excluding emergency observation arranged for routine infusion or examination) and unplanned readmissions (defined as readmissions due to disease progression, treatment-related adverse reactions, or any other unplanned reasons within 30 days after discharge).

#### 2.5.6 High intensity medical use

A key indicator for evaluating the quality of end-of-life care. Including: ① The proportion of patients who received any systemic chemotherapy (including targeted therapy and immunotherapy) within 30 days before their death; ② The proportion of patients newly admitted to the intensive care unit (ICU) within 14 days before their death; ③ The total length of hospital stay during the entire observation period.

#### 2.5.7 Palliative treatment intervention

Record the number of interventional procedures received by patients aimed at relieving symptoms rather than curing the tumor. Including: ① the number of palliative radiotherapy sessions for painful bone metastases or other symptomatic lesions; ② The number of palliative puncture drainage procedures performed to alleviate pleural effusion, peritoneal effusion, or pericardial effusion; ③ The number of palliative stent placement or ostomy procedures performed to relieve gastrointestinal obstruction.

#### 2.5.8 Survival outcome: overall survival

Defined as the period from the start of the first systematic anti-tumor treatment (index date) to the date of death for any reason or the last effective follow-up date (if the patient survived until the cutoff date). Survival status is confirmed through electronic medical record systems, outpatient follow-up records, and telephone follow-up (only used to confirm death status).

### 2.6 Statistical methods

All statistical analyses were based on two-sided tests, with P<0.05 indicating statistically significant differences. Categorical variables are expressed as examples (percentages), and between group comparisons are conducted using chi square test or Fisher’s exact test (when expected frequency<5). The normality test for continuous variables is conducted using the Shapiro Wilk test. Continuous variables that follow a normal distribution are represented by mean ± standard deviation, and independent sample t-test is used for inter group comparison; Non normally distributed continuous variables are represented by the median (interquartile range), and Mann Whitney U test is used for inter group comparisons. The main effects analysis used multivariate logistic regression (for binary outcomes) or generalized linear models (for skewed continuous outcomes), reporting adjusted odds ratios (aOR) or geometric mean ratios and their 95% CI. Survival analysis used Kaplan Meier method to plot survival curves, inter group comparisons used Log rank test, and Cox proportional hazards model was constructed for multivariate adjustment, reporting hazard ratio (HR) and 95% CI. All models were required to test the proportional hazards hypothesis (for Cox models) or model goodness of fit. The competitive risk analysis adopts the Fine Gray model and reports the subdistribution hazard ratio (SHR). All data processing and statistical analysis were completed in SPSS 26.0 and R 4.2.1 software.

## 3 Results

### 3.1 Baseline characteristics of patients

Firstly, all outpatient and inpatient patients diagnosed with colon or rectal malignant tumors (n = 1800) are retrieved from the electronic medical record system through ICD-10 coding (C18-C20) and diagnostic description. Subsequently, according to the AJCC 8th edition staging criteria, cases diagnosed as stage IV (metastatic) were screened out (n = 600). Further exclude patients who give up treatment before the first systemic treatment, only receive optimal supportive therapy (n = 50), and patients who participate in other interventional clinical studies at the same time (n = 30). Finally, cases with more than 20% missing key variables in the medical records (n = 15) were excluded, and data integrity verification was performed on the remaining cases, resulting in the inclusion of approximately 205 patients for statistical analysis(**Figure 1**). This study initially included 205 patients, of which 4 (1 in the combination group and 3 in the non-combination group) were excluded from the final analysis due to loss to follow-up (inability to confirm survival status). The actual analyzed population was 201 (69 in the combination group and 132 in the non-combination group), with a loss to follow-up rate of 2.0%.

The comparison of baseline characteristics between the combined and non-combined groups of metastatic colorectal cancer patients showed statistical differences in age (Z = 3.046, P = 0.002), ECOG PS score distribution (χ ²=6.769, P = 0.009), Charlson comorbidity index (Z = 4.545, P<0.001), and peritoneal metastasis rate (χ ²=4.667, P = 0.031). There were no significant differences (P>0.05) between the two groups in terms of gender, primary site, liver metastasis, lung metastasis, other metastases, and the number of metastatic sites. There was no statistically significant difference in the distribution of first-line treatment plans (χ ²=4.370, P = 0.112). See **Table 1**.

**Table 1:** Comparison of baseline characteristics of patients with metastatic colorectal cancer.

Characteristic	Combined group (n=69)	Non-combined group (n=132)	Statistic	P-value
Age, median (IQR)	68.4 (61.2, 74.8)	63.7 (56.5, 71.3)	Z=3.046	0.002
Sex, n (%)			χ²=0.116	0.734
Male	38 (55.1)	76 (57.6)		
Female	31 (44.9)	56 (42.4)		
Primary Tumor Site, n (%)			χ²=0.047	0.829
Colon	45 (65.2)	88 (66.7)		
Rectum	24 (34.8)	44 (33.3)		
Liver Metastasis	42 (60.9)	85 (64.4)	χ²=0.234	0.629
Lung Metastasis	31 (44.9)	52 (39.4)	χ²=0.574	0.449
Peritoneal Metastasis	28 (40.6)	34 (25.8)	χ²=4.667	0.031
Other Metastasis	19 (27.5)	41 (31.1)	χ²=0.270	0.603
Number of Metastatic Sites, median (IQR)	2.0 (2.0, 3.0)	2.0 (1.0, 3.0)	Z=1.520	0.128
ECOG PS, n (%)			χ²=6.769	0.009
0-1	37 (53.6)	95 (72.0)		
≥2	32 (46.4)	37 (28.0)		
CCI, median (IQR)	6.0 (4.0, 8.0)	4.0 (2.0, 6.0)	Z=4.545	<0.001
First-Line Regimen, n (%)			χ²=4.370	0.112
Chemotherapy ± Bevacizumab	41 (59.4)	58 (43.9)		
Chemotherapy + anti-EGFR	18 (26.1)	49 (37.1)		
Immunotherapy	10 (14.5)	25 (19.0)		

*anti-EGFR: Cetuximab or Panitumumab. **Used only for dMMR/MSI-H patients.

### 3.2 Patient reported outcomes and treatment decision indicators

**Figure 2:**
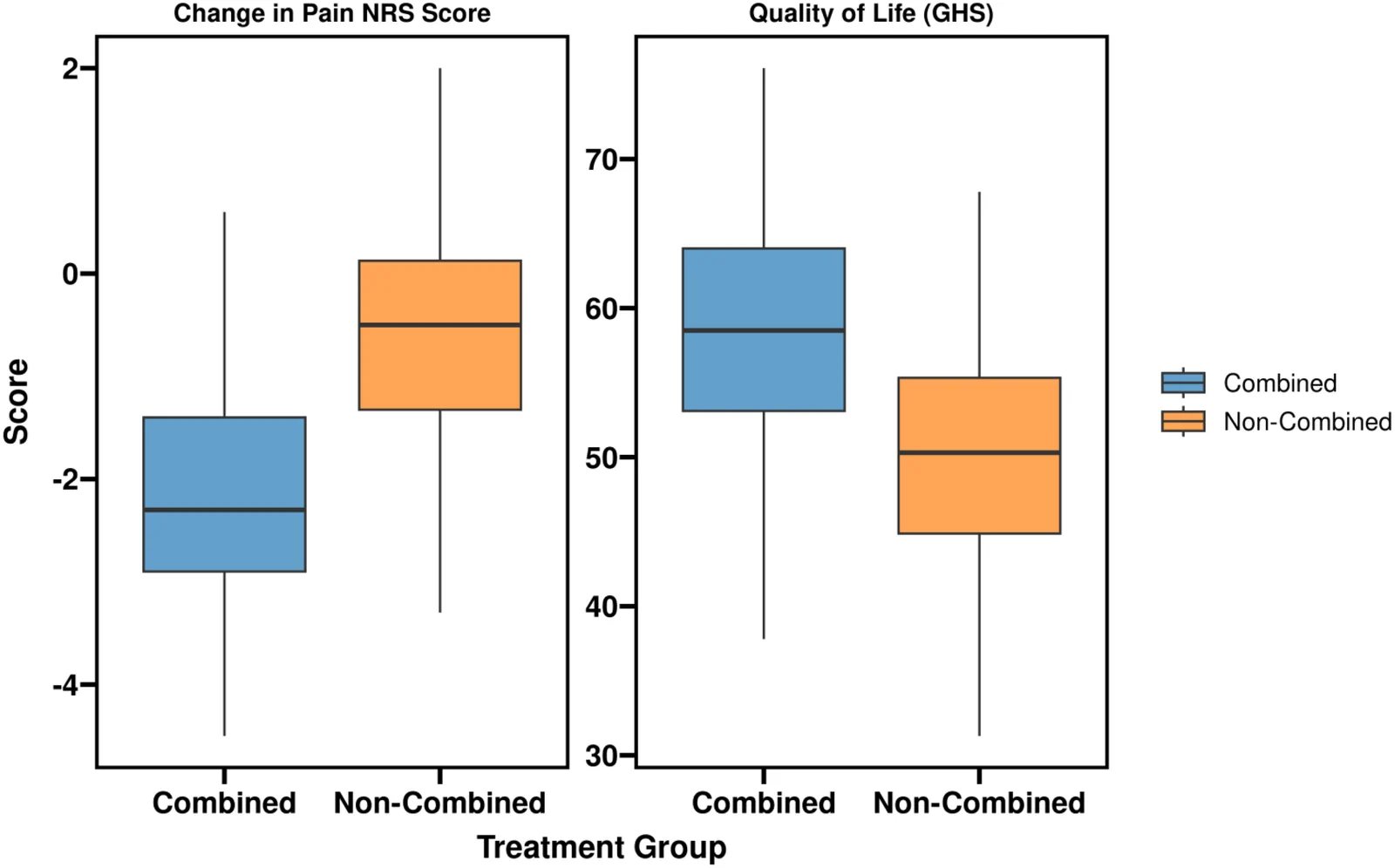
Comparison of NRS and GHS. Patients with available EORTC QLQ-C30 Global Health Status data (n=112) did not differ significantly from those without (n=89) in terms of age (P = 0.41), sex (P = 0.68), ECOG PS ≥2 (P = 0.53), CCI (P = 0.37), or peritoneal metastasis (P = 0.59), suggesting that missing QoL data were not systematically differential by these measured characteristics.

The comparison of patient-reported outcome indicators is presented in **Table 2**. For the primary outcome of pain, the median between-group difference (Hodges–Lehmann estimate) in NRS change was −1.5 (95% CI −2.0 to −1.0; P<0.001). Treatment goal concordance, the second primary outcome, was 37.6 percentage points higher in the combined group (95% CI 26.5% to 48.7%; P<0.001). Among secondary outcomes, clinically documented emotional distress was 19.5 percentage points lower (95% CI −33.0% to −6.0%; P = 0.008), and the global health status/QoL score showed a median improvement of 8.3 points (95% CI 4.0 to 12.5; P<0.001). The distribution of individual pain NRS changes is shown in **Figure 2**.

**Table 2:** Comparison of patient-reported outcomes and treatment decision metrics.

Indicator	Combined group (n=69)	Non-combined group (n=132)	Effect estimate (95% CI)	Statistic	P-value
Baseline Pain NRS, median (IQR)	6 (5, 7)	5 (4, 6)	1 (0 to 1)	Z=3.046	0.002
Change in Pain NRS, median (IQR)	-2.0 (-3.5, -1.0)	-0.5 (-1.5, 0.5)	−1.5 (−2.0 to −1.0)	Z=-5.825	<0.001
Clinically documented emotional distress, n (%)	19 (27.5)	62 (47.0)	−19.5% (−33.0% to −6.0%)	χ²=7.120	0.008
Baseline EORTC QLQ-C30 GHS, median (IQR)*	45.8 (41.7, 54.2)	45.8 (37.5, 54.2)	0 (−4.2 to 4.2)	Z=-0.41	0.68
Follow-up EORTC QLQ-C30 GHS, median (IQR)*	58.3 (50.0, 66.7)	50.0 (41.7, 58.3)	8.3 (4.0 to 12.5)	Z=4.025	<0.001
Treatment Goal Concordance, n (%)	62 (89.9)	69 (52.3)	37.6% (26.5% to 48.7%)	χ²=28.423	<0.001

*EORTC QLQ-C30 Global Health Status subscale score, range 0-100, higher scores indicate better outcomes. The EORTC QLQ-C30 Global Health Status subscale was available for 112 patients (42 in the combined group, 70 in the non-combined group), representing 55.7% of the study population.

### 3.3 Overall survival analysis

**Figure 3:**
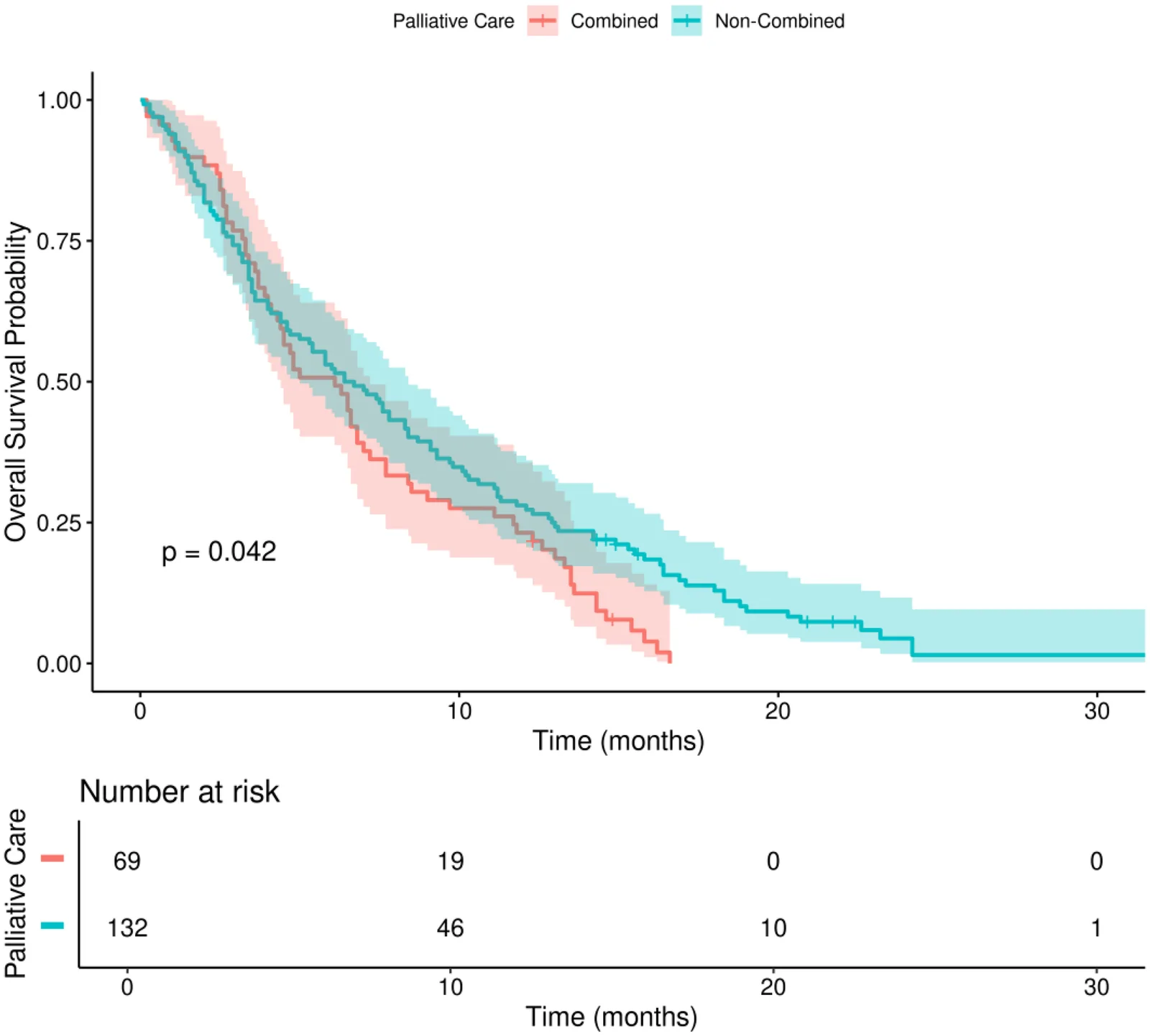
Overall survival analysis.

A total of 156 patients (77.6%) died. The median follow-up time was 18.5 months (IQR: 12.2-24.8). Kaplan Meier survival analysis showed no significant difference in overall survival (OS) between the combined palliative care group and the non-combined group (median OS: 19.1 months vs. 18.3 months); Risk ratio HR = 0.87, 95% CI 0.63-1.21; Logarithmic rank test P = 0.412) (**Figure 3**).

### 3.4 Multivariate regression analysis

In multivariate regression analysis, outcome variables include changes in pain NRS score (continuous variable), clinically documented emotional distress (binary, = 1), treatment goal consistency (binary, = 1), unplanned readmission frequency (count variable), and chemotherapy 30 days before death (binary, = 1). After adjusting for confounding factors, the combination of palliative care support was an independent influencing factor for pain improvement (β=-1.42, P<0.001), reduced risk of clinically documented emotional distress (aOR=0.42, P = 0.008), improved treatment goal consistency (aOR=7.85, P<0.001), reduced unplanned readmission (IRR = 0.66, P = 0.001), and reduced 30 day chemotherapy before death (aOR=0.26, P<0.001). See **Table 3**. Multivariate Cox regression analysis of overall survival showed that palliative care support (yes vs no) did not significantly affect survival risk (HR = 0.92, P = 0.632). ECOG PS score ≥ 2 (HR = 1.82, P<0.001) and increased Charlson comorbidity index score (HR = 1.08, P = 0.021) are independent influencing factors for increased survival risk. There was no independent statistical association between age, peritoneal metastasis, first-line treatment regimen, and survival risk (all P>0.05). See **Table 4**.

**Table 3:** Multivariable regression analysis: impact of palliative care support on key outcomes.

Outcome variable	Model type	Regression coefficient (β) or adjusted odds ratio (aOR)	95% Confidence interval	P-value
Change in Pain NRS Score	Linear Regression	β = -1.42	(-2.01, -0.83)	<0.001
clinically documented emotional distress (Yes vs. No)	Logistic Regression	aOR = 0.42	(0.22, 0.80)	0.008
Treatment Goal Concordance (Yes vs. No)	Logistic Regression	aOR = 7.85	(3.52, 17.52)	<0.001
Number of Unplanned Readmissions	Negative Binomial Regression	IRR* = 0.66	(0.51, 0.85)	0.001
Chemotherapy within Last 30 Days of Life (Yes vs. No)	Logistic Regression	aOR = 0.26	(0.12, 0.57)	<0.001

*IRR, Incidence Rate Ratio; indicating the ratio of the incidence rate in the combined group relative to the non-combined group. All models were adjusted for age, sex, ECOG PS (≥2 vs. 0-1), CCI, peritoneal metastasis (yes/no), and first-line treatment regimen.

**Table 4:** Multivariable Cox proportional hazards regression analysis for overall survival (OS).

Variable	Hazard ratio (HR)	95% Confidence interval	P-value
Palliative Care Support (Yes vs. No)	0.92	0.65 - 1.30	0.632
Age (per 10-year increase)	1.18	0.98 - 1.42	0.078
ECOG PS (≥2 vs. 0-1)	1.82	1.32 - 2.51	<0.001
CCI (per 1-point increase)	1.08	1.01 - 1.15	0.021
Peritoneal Metastasis (Yes vs. No)	1.25	0.89 - 1.76	0.195
First-line regimen (reference: chemotherapy)			
Chemotherapy + anti-EGFR	0.85	0.59 - 1.23	0.388
Immunotherapy	0.62	0.37 - 1.04	0.071

### 3.5 Medical resource utilization

The between-group comparisons for pre-specified medical resource utilization indicators are summarized in **Table 5**. Patients in the combined palliative care group had significantly fewer unplanned readmissions (median 1 [IQR 0–2] vs. 2 (1–3); median difference −1, 95% CI −1 to 0; P<0.001) and fewer emergency department visits (median 0 (0–1) vs. 1 (0–2); median difference −1, 95% CI −1 to 0; P<0.001). Among the 156 patients who died during the study period, the proportion receiving systemic chemotherapy within 30 days of death was substantially lower in the combined group (13.5% vs. 40.4%; rate difference −26.9%, 95% CI −40.1% to −13.7%; P<0.001), as was the proportion with a new ICU admission within 14 days of death (5.8% vs. 17.3%; rate difference −11.5%, 95% CI −21.9% to −1.1%; P = 0.031). Total inpatient length of stay did not differ significantly between groups (median 21 days [IQR 14–31] vs. 24 days (16–35); P = 0.22). The overall utilization of palliative interventions (palliative radiotherapy, drainage procedures, stent/ostomy placement) was similar between groups (26.1% vs. 18.9%; P = 0.23).

**Table 5:** Comparison of medical resource utilization indicators.

Indicator	Combined PC group (n=69)	Non-combined PC group (n=132)	Effect estimate (95% CI)	P-value
Number of unplanned readmissions, median (IQR)	1 (0, 2)	2 (1, 3)	−1 (−1 to 0)	<0.001
Number of ED visits, median (IQR)	0 (0, 1)	1 (0, 2)	−1 (−1 to 0)	<0.001
Chemotherapy within 30 days of death, n (%)	7/52 (13.5)	42/104 (40.4)	−26.9% (−40.1% to −13.7%)	<0.001
ICU admission within 14 days of death, n (%)	3/52 (5.8)	18/104 (17.3)	−11.5% (−21.9% to −1.1%)	0.031
Total inpatient length of stay, days, median (IQR)	21 (14, 31)	24 (16, 35)	−3 (−8 to 2)	0.22
Any palliative intervention, n (%)	18 (26.1)	25 (18.9)	7.2% (−4.9% to 19.2%)	0.23

### 3.6 Treatment-related adverse events

There was no statistically significant difference in the incidence of treatment-related adverse events between the two groups at or above level 3. The inter group comparison P values for any grade ≥ 3 adverse events (P = 0.586), neutropenia (P = 0.949), anemia (P = 0.560), diarrhea (P = 0.679), fatigue (P = 0.518), nausea/vomiting (P = 0.910), hypertension (P = 0.632), and dose adjustment/delay due to adverse events (P = 0.284) are all greater than 0.05. See **Table 6**.

**Table 6:** ≥ grade 3 treatment-related adverse events.

Adverse event, n (%)	Combined palliative care group (n=69)	Non-combined palliative care group (n=132)	P-value
Any ≥ Grade 3 AE	44 (63.8)	79 (59.8)	0.586
Hematologic toxicity			
Neutropenia	18 (26.1)	35 (26.5)	0.949
Anemia	8 (11.6)	12 (9.1)	0.56
Non-hematologic toxicity			
Diarrhea	12 (17.4)	20 (15.2)	0.679
Fatigue	10 (14.5)	15 (11.4)	0.518
Nausea/Vomiting	5 (7.2)	9 (6.8)	0.91
Hypertension	4 (5.8)	10 (7.6)	0.632
Dose Adjustment/Delay due to AE	25 (36.2)	38 (28.8)	0.284

*Graded according to CTCAE v5.0. AE, Adverse Event.

### 3.7 Competitive risk and subgroup analysis

#### 3.7.1 Competitive risk analysis

The Fine-Gray competing risk model for chemotherapy within 30 days of death included the same set of covariates as the primary multivariable analyses (age, ECOG PS ≥2, CCI, peritoneal metastasis, and first-line treatment regimen), with death treated as a competing event. The results confirmed that the combination of PC significantly reduced the risk of this event (subdistribution hazard ratio SHR = 0.31, 95% CI 0.16–0.60, P<0.001).

#### 3.7.2 Subgroup analysis

Subgroup analysis was conducted by age (<70/≥ 70 years), ECOG PS (0-1/≥ 2), and primary site (colon/rectum). The interaction tests showed no statistically significant heterogeneity in the associations of PC with pain control improvement (all interaction P>0.20) or reduction of chemotherapy in the last 30 days of life (all interaction P>0.15) across prespecified subgroups. However, given the limited sample size and low statistical power for detecting interactions, the absence of significant heterogeneity does not demonstrate homogeneous treatment effects and should be interpreted with caution.

## 4 Discussion

The treatment goal of metastatic colorectal cancer (mCRC) has evolved from simply pursuing survival extension to maximizing quality of life, managing symptoms, and ensuring treatment decisions are consistent with patient wishes while extending survival (16, 17). This study explored the impact of integrating professional palliative care support on multidimensional outcomes of mCRC patients on the basis of standard systemic therapy through retrospective cohort analysis. From the data, joint palliative care support is significantly correlated with better pain control, lower incidence of clinically documented emotional distress, higher consistency in treatment decisions, and more rational utilization of end-of-life medical resources, although there is no difference in overall survival between the two groups. Notably, the between-group difference in pain NRS change (median −1.5 points) was statistically significant but falls below the conventional MCID threshold of 2 points for cancer-related pain. However, in the context of the combined group’s higher baseline pain severity (median NRS 6 vs. 5), a 2-point within-group reduction represents a clinically meaningful improvement for individual patients. Similarly, the 8.3-point improvement in EORTC QLQ-C30 Global Health Status/QoL in the combined group exceeds the established MCID of 5–10 points for this scale, suggesting a clinically relevant benefit in perceived quality of life.

These findings provide strong evidence from the real world for integrating multidisciplinary palliative care in the early stages of advanced cancer treatment. The mechanism behind the improvement in patient reported outcomes, especially the significant advantage in pain control, deserves further exploration (18). On the one hand, the professional evaluation and medication titration of complex cancer pain by palliative care teams may directly contribute to the decline in pain scores (19). On the other hand, the reduction of clinically documented emotional distress suggests that psychosocial support plays a crucial role. Chronic stress and anxiety can lower pain thresholds and exacerbate pain, while the psychological counseling and coping strategies provided by palliative care may indirectly improve the pain experience by alleviating clinically documented emotional distress (20). This study observed that the combined group had fewer records of clinically documented emotional distress diagnosis or medication use, which is consistent with the practice model of early intervention and active handling of psychosocial issues by the team (21).

In terms of treatment decision-making and medical resource utilization, the results of this study present a clear logical chain. The joint palliative care group showed a high degree of consistency in treatment goals (89.9%), which may be closely related to the team’s systematic discussion of “advance care plans” (22). Through repeated communication, help patients and their families clarify treatment priorities (such as the balance between quality of life and survival), making subsequent clinical decisions (such as whether to use new line chemotherapy or transfer to ICU) more likely to be in line with the patient’s original intention (23).

The prepositioning and transparency of this decision-making process directly explains why the proportion of patients in the combined group receiving high-intensity medical treatment (such as chemotherapy and ICU admission) before their death is significantly lower. From a resource perspective, reducing these interventions that may go against the wishes of patients and have minimal benefits is not only ethical, but may also alleviate the burden on the healthcare system (24). Although this study did not directly measure cost-effectiveness (25).

Place this study in a broader literature context for examination. The evidence on the impact of palliative care on the utilization of medical resources is not uniform (26). Partial randomized controlled trials support that early palliative care can reduce emergency and hospitalization (27), but there are also observational studies that have not found this effect (28). In this study, the combination group showed advantages in unplanned readmissions and emergency visits, but there was no difference in total length of hospital stay. This cannot be ignored (29). One reasonable speculation is that palliative care has reduced unplanned hospitalizations due to uncontrolled acute symptoms through better symptom management and family support; However, the impact on planned hospitalization or palliative procedures required for disease progression itself is limited (30).

It is noteworthy that although patients in the PC group had significantly worse baseline prognostic features—older age, higher ECOG PS, greater comorbidity—their overall survival was similar to that of the non-PC group (adjusted HR 0.92, 95% CI 0.65–1.30). This finding could be interpreted in two ways: either PC integration does not influence survival, or the lack of difference reflects a potential survival benefit that was counterbalanced by the higher baseline risk. The observational nature of our study precludes definitive conclusions. Nevertheless, the consistent improvements in symptom control, treatment goal concordance, and end-of-life care quality suggest that the primary value of PC in this context lies in enhancing the quality and patient-centeredness of care.

This study has several limitations. First, because of the retrospective, non-randomized design, patients who received palliative care (PC) had substantially worse baseline prognostic characteristics, including older age, poorer performance status, higher comorbidity burden, and more frequent peritoneal metastases. Although we performed multivariable adjustment and propensity score matching to reduce confounding by measured variables, these methods cannot fully eliminate residual confounding from unmeasured or unknown factors. Therefore, all observed associations should be interpreted cautiously as hypothesis-generating only, and no causal inference regarding the effect of PC should be drawn. Secondly, some patients’ reported outcomes rely on medical records, which may lead to underestimation. Furthermore, EORTC QLQ-C30 scores were available for only 55.7% of the study population. Although patients with and without available data did not differ significantly on measured baseline characteristics, unmeasured factors affecting QoL completion (e.g., rapid clinical deterioration) may still introduce selection bias and limit the generalizability of the quality-of-life findings. Finally, this study was conducted in a single center, and the patient population and palliative care practice model may have specificity, so extrapolation of conclusions should be cautious. Second, the binary definition of PC exposure based on any formal consultation is inherently broad. Data on the frequency, cumulative duration, and timing of PC consultations were not systematically recorded, precluding any dose–response analysis. A single consultation and sustained multidisciplinary follow-up represent very different intensities of intervention, and the binary classification may have diluted the true effect size or obscured heterogeneity of benefit. Furthermore, the exact timing of the first PC consultation relative to systemic therapy initiation was incompletely documented, which prevented a formal comparison of early versus late integration. These limitations regarding PC exposure characterization should be addressed in future prospective studies with prospective, protocolized recording of PC delivery. Although a sensitivity analysis excluding patients who died within 90 days yielded consistent results, residual immortal time bias cannot be excluded because PC exposure was analyzed as a fixed baseline characteristic. Patients with longer survival had greater opportunity to receive PC, which may still inflate the apparent benefits despite the exclusion of early deaths. A time-dependent exposure analysis, which would more appropriately account for the timing of PC initiation, was not feasible with our data. Consequently, the magnitude of the observed associations may be overestimated, and the results should be interpreted with even greater caution. The lack of detailed timing data also precluded an analysis of early versus late PC integration, which should be addressed in future prospective studies. The inability to analyze PC timing limits our capacity to differentiate the effects of early integration from those of late referrals, which may differ substantially. The definition of clinically documented emotional distress relied on clinical documentation and medication prescriptions, which likely captures only moderate-to-severe distress. Milder distress and untreated psychological symptoms may have been under-ascertained, a limitation inherent to retrospective designs. Subgroup analyses were exploratory and may have been underpowered due to the relatively small number of patients in the PC group (n=69); non-significant interaction tests should not be interpreted as evidence of homogeneous effects. Propensity score matching was a *post hoc* analysis added to further evaluate the robustness of the primary findings. Subgroup analyses, although prespecified, were exploratory and underpowered; the absence of statistically significant interactions should not be interpreted as evidence of homogeneous treatment effects. Additionally, multiple secondary and subgroup endpoints were evaluated without adjustment for multiplicity. The risk of type I error is therefore inflated, and findings for secondary outcomes and subgroup analyses should be considered exploratory and hypothesis-generating rather than confirmatory.

In summary, in this retrospective cohort study, concurrent professional PC was associated with better symptom control, higher treatment-goal concordance, and less aggressive end-of-life care, without a statistically significant difference in overall survival. Given the observational design and the potential for residual confounding, these associations should not be interpreted as causal. While the findings support the integration of PC into standard mCRC care as a hypothesis-generating real-world observation, prospective randomized studies are required to establish causality and inform clinical practice.

## Data Availability

The original contributions presented in the study are included in the article/**Supplementary Material**. Further inquiries can be directed to the corresponding author.

## Supplementary material

Supplementary Table 1 Sensitivity analysis of key outcomes after excluding patients who died within 90 days of systemic therapy initiation (immortal time bias assessment). PC, palliative care; NRS, Numerical Rating Scale; IQR, interquartile range. From the full analysis set (n=201), 24 patients who died within 90 days of starting first-line systemic therapy were excluded (4 in the combined group, 20 in the non-combined group).
