# Supplementary material for: Comparison of patient-reported outcomes and healthcare utilization between metastatic colorectal cancer patients receiving systemic therapy with or without concurrent palliative care support: a retrospective cohort study
Source: Front Oncol. 2026 Sep 3;16:1882581. doi: 10.3389/fonc.2026.1882581 (PMC13581539; doi:10.3389/fonc.2026.1882581)
Supplement: Supplementary file 1 [file Table1.docx]

Supplementary Table S1. Sensitivity analysis of key outcomes after excluding patients who died within 90 days of systemic therapy initiation (immortal time bias assessment).

| Outcome | Combined PC Group (n=65) | Non-Combined Group (n=112) | Statistic | P-value |
| --- | --- | --- | --- | --- |
| Change in Pain NRS, median (IQR) | -2.0 (-3.5, -1.0) | -0.5 (-1.5, 0.5) | Z = -5.62 | <0.001 |
| Clinically documented clinically documented emotional distress, n (%) | 18 (27.7) | 51 (45.5) | χ² = 5.16 | 0.023 |
| Treatment goal concordance, n (%) | 58 (89.2) | 63 (56.3) | χ² = 20.14 | <0.001 |
| Chemotherapy within 30 days of death, n (%) | 6 (9.2) | 31 (27.7) | χ² = 8.02 | 0.005 |
| Number of unplanned readmissions, median (IQR) | 1 (0, 2) | 2 (1, 3) | Z = -2.98 | 0.003 |

Abbreviations: PC, palliative care; NRS, Numerical Rating Scale; IQR, interquartile range.

From the full analysis set (n=201), 24 patients who died within 90 days of starting first-line systemic therapy were excluded (4 in the combined group, 20 in the non-combined group).
